# Postoperative pain management: Comparison of intravenous paracetamol versus ketorolac

**DOI:** 10.6026/973206300214673

**Published:** 2025-12-15

**Authors:** Amit Kumar Prasad, Rajesh Kumar Jha, Asim Shekhar

**Affiliations:** 1 Department of Anaesthesiology, RDJM Medical College and Hospital, Muzaffarpur, Bihar, India

**Keywords:** Postoperative pain, intravenous paracetamol, intravenous ketorolac, non-opioid analgesics, visual analogue scale, abdominal surgery

## Abstract

Effective control of postoperative pain remains a key challenge in perioperative care, necessitating comparison of commonly used analgesic agents. Therefore, it is of interest to compare the analgesic efficacy of intravenous paracetamol and intravenous ketorolac in 60 patients undergoing elective surgery. Patients received either paracetamol 1 g or ketorolac 30 mg intravenously, and pain intensity was assessed using the Visual Analogue Scale with documentation of rescue analgesic use. Ketorolac produced significantly lower pain scores and reduced need for rescue analgesia compared with paracetamol (p < 0.05). Thus, we show that intravenous ketorolac provides superior postoperative analgesia within a multimodal pain management strategy.

## Background:

Effective management of postoperative pain is an essential aspect of enhanced recovery following surgery, as uncontrolled pain delays mobilization, prolongs hospitalization, increases morbidity and diminishes patient satisfaction [1]. Opioids have long been the standard agent for postoperative analgesia due to their potent effect. However, their use is limited by a range of well-known side effects, including respiratory depression, sedation, nausea, vomiting and development of tolerance, constipation and potential for dependence [2, 3]. These issues have driven interest into alternative or adjunct non-opioids agents for use, particularly as part of multimodal pain management regimens. Paracetamol (acetaminophen) and non-steroidal anti-inflammatory drugs (NSAIDs) are some of the most common non-opioid agents that are utilized because they have favorable safety profiles and opioid-sparing effect [4]. Intravenous paracetamol works centrally by inhibiting cyclooxygenase (COX) activity in the brain and modulating serotonergic and cannabinoid pathways to decrease pain perception, with little peripheral anti-inflammatory effects [5, 6]. Paracetamol is usually well tolerated, gives little to no adverse gastrointestinal or renal function and does not compromise platelet function seen with NSAIDs. Ketorolac is another potent NSAID to provide analgesia through non-selective inhibition of COX-1 and COX-2, thereby reducing prostaglandin synthesis [7]. Its effectiveness for moderate postoperative pain is often comparable to opioids, while having additional anti-inflammatory efficacy. However, side effects, such as gastric irritation, renal failure and bleeding risk from platelet inhibition, constrain the use of ketorolac in some populations [8]. Nevertheless, ketorolac is widely utilized and maintained its effectiveness as an analgesic when given according to the recommended duration and dosage. Several clinical trials and systematic reviews have compared ketorolac to intravenous paracetamol among adult patients, with mixed results regarding which option was more effective depending on the patient population, type of surgery and method of assessing outcomes [9, 10]. The preemptive use of IV ketorolac provided better postoperative analgesia compared with IV paracetamol in patients undergoing elective laparoscopic surgeries [11]. Some studies indicated that ketorolac produced superior analgesia; other studies using different surgical populations did not find the analgesia between the two agents to be different. It is acknowledged there is limited data regarding paracetamol and ketorolac in certain surgical populations in our regional context; therefore, it was essential to evaluate both agents to better understand the use of both medications for postoperative pain relief. Therefore, it is of importance to compare intravenous paracetamol to intravenous ketorolac in patients undergoing elective surgery, with a primary aim to measure postoperative pain scores and a secondary aim to evaluate the need for rescue analgesia.

## Materials and Methods:

This was a randomized controlled comparative trial performed in Department of Anaesthesiology, RDJM Medical College and Hospital, Muzaffarpur, Bihar, India for one year after obtaining approval from the Institutional Ethics Committee and obtaining informed written consent from patients included in the study. Sixty patients of either sex, between the ages of 18 and 60 years, posted for elective abdominal surgeries under general anaesthesia were included in the study. Patients with a prior history of hypersensitivity to paracetamol or NSAIDs, bleeding disorders, peptic ulcer diseases, hepatic or renal dysfunction and/or patients using anticoagulant/ antiplatelet therapy at admission were excluded from the study. Anaesthetic management was standardized in all patients and patients were randomized to two equal groups of thirty using a computer-generated randomization table. Group P was given intravenous paracetamol 1 g (100 ml) over 15 minutes. Group K was given intravenous ketorolac 30 mg in 100 ml normal saline over 15 minutes. The study medications are given at the end of surgery once hemodynamic stability was attained. Postoperative pain was measured using a ten-point Visual Analogue Scale (VAS), which indicated 0 as no pain and 10 as worst pain imaginable. A blinded assessor conducted assessments postoperatively at 0, 1, 2, 4, 6 and 12 hours. For rescue analgesia injection tramadol 50 mg was given as intravenous slow infusion, when the patient had a VAS score of at least 4. Total numbers of rescue analgesia given over 12 hours were recorded. Any adverse events (nausea/vomiting, gastric irritation or bleeding tendency) were documented as well. Data were consolidated in Microsoft Excel and analyzed using SPSS (version 26.0; IBM Corp., Armonk, NY, USA). Continuous variables (age) were presented as mean ± standard deviation and were compared using Student's t-test. Categorical variables (sex distribution, adverse events) were analyzed using chi-square. Any p-values of < 0.05 were considered statistically significant.

## Results:

All sixty participants completed the study without any deviations or dropouts from the protocol. The demographic variables, including average age, sex and type of surgical procedures was similar between both the groups, suggesting that the randomization process had generated balanced baseline characteristics in the two groups. Patients from both groups reported increasing pain scores over time postoperatively, assessed using the Visual Analogue Scale (VAS). However, patients receiving ketorolac were consistently reporting lower pain scores than paracetamol, with statistically significant differences at 2, 4, 6 and 12 hours postoperatively. A large number of patients in the paracetamol group reported moderate to severe pain requiring additional rescue analgesia, whereas fewer patients in the ketorolac group required rescue analgesia. The total number of rescue doses of rescue analgesia was also much higher in the paracetamol group. However, mild adverse events occurred in both groups. The ketorolac group reported a higher frequency and incidence of nausea, vomiting and gastric discomfort, however, this was not statistically significant. There were no serious adverse events, including no incidence of complications from surgery like excessive bleeding, renal failure, or allergic reactions to medications were reported in either group during the study. The evidence supports that both IV paracetamol and ketorolac are safe and effective options for the treatment of postoperative pain although ketorolac was associated with superior analgesia and lower use of rescue analgesia. This would suggest that ketorolac may be a useful agent for treating moderate postoperative pain but should be prescribed with monitoring for gastrointestinal adverse effects in usual practice. Table 1 (see PDF) confirmed that both groups were comparable in terms of age, sex and surgical distribution, ensuring a balanced baseline. Table 2 (see PDF) demonstrated consistently lower VAS pain scores in the ketorolac group, with significant differences noted from the 2-hour interval onwards. Table 3 (see PDF) highlighted that a significantly greater number of patients in the paracetamol group required rescue analgesia, both in frequency and total doses. Table 4 (see PDF) showed that adverse events were infrequent in both groups, with ketorolac causing slightly higher gastrointestinal discomfort, though without statistical significance. Table 5 (see PDF) consolidated the outcomes, confirming the superior analgesic efficacy of ketorolac while demonstrating that both drugs maintained acceptable safety profiles.

## Discussion:

This randomized comparative study shows that intravenously administered ketorolac provides better early postoperative analgesia in comparison with intravenously administered paracetamol, as demonstrated by a lower mean VAS from the 2 hours timepoint and significantly less usage of rescue analgesia (23.3 % v 60.0 %, p = 0.004). Adverse events for both agents were generally well tolerated and no major adverse events occurred; however, ketorolac group had small and statistically nonsignificant increases in gastrointestinal discomfort. These findings support the use of ketorolac as an efficacious non-opioid analgesic for immediate postoperative pain in patients with no contraindications. The ketorolac group experienced lower pain scores than the paracetamol group from 2-12 hours which is consistent with previous randomized trials and meta-analyses showing greater analgesic efficacy for NSAIDs compared with paracetamol for moderate-severe postoperative pain, particularly early in the postoperative period when inflammatory mediators are likely influencing nociception [12]. This mechanism of action is due largely to non-selective inhibition of the cyclooxygenase isoforms and peripheral suppression of the synthesis of prostaglandins, inducing both analgesia and anti-inflammatory responses which are advantageous in the acute postoperative period [13]. In contrast, paracetamol appears to operate primarily centrally and is unlikely to have a peripheral anti-inflammatory mechanism of action that may explain the higher pain scores and greater use of rescue analgesia in the paracetamol group [14]. The clinical implications of these results in terms of opioid-sparing are meaningful. Ketorolac, in helping to avoid rescue analgesia, forms an aspect of multimodal analgesia regimens that seeks to maximize the use of non-opioid drugs while decreasing side effects on patients (nausea, sedation, respiratory depression and others) associated with treating with opioid medications, a consideration that has contributed to increased use of non-opioid adjuncts in perioperative care [15]. Similar to recommendations previously published, we advocate for the inclusion of non-steroidal anti-inflammatory drugs (NSAIDs) in multimodal regimens for treatment where not contraindicated, as early postoperative analgesia can help with early mobilization and achieve better recovery - or at the very least, a more comfortable recovery for patients [16]. In considerations of safety in using ketorolac, only mild gastric irritation was recorded in a small number of patients in this study. The risks of using a NSAID (gastrointestinal bleeding, platelet dysfunction and renal dysfunction) warrants patient selection, short course (generally ≤ 5 days) and monitoring in patients at risk [17]. Paracetamol remains an important analgesic option for patients with contraindications to NSAIDs (peptic ulcer disease, bleeding diathesis and significant renal dysfunction) and has excellent safety profile both gastrointestinal and in terms of renal function [18]. Therefore, the clinician is generally in a position to weigh pain relief and treatment strength against pain morbidity, long term comorbidity and bleeding risk in an individual patient. The methodological characteristics of this study bolstered the validity of its conclusions. Random assignment and comparable initial demographics reduced selection bias; the postoperative assessments were conducted by blinded observers at pre-defined times using a validated VAS instrument; and the requirement for rescue analgesia (VAS ≥4) is an objective threshold relevant to clinical practice. Additionally, the daily dosing regimen (every 6 hours for 24 hours) exemplified common practice in the postoperative period and allowed for the analysis of analgesic effect during the critical first postoperative day [19]. Nonetheless, several limitations warrant caution in generalizing findings. The sample size was modest (n=60), limiting power to detect less familiar side effects and subgroup effects; the participants were a heterogeneous sample with regard to surgical procedures (elective abdominal surgeries), which may modulate pain trajectories and pain responsiveness to analgesics; and the observation period (12 hours for primary outcomes, 24 hours for dosing) captured early analgesic differences but reliant on short-term metrics did not account for longer-term pain control or functional recovery. Furthermore, while tramadol rescue is a practical clinical outcome, documenting total opioid use in morphine equivalents would be a more standardized approach to assess opioid-sparing benefit. Finally, the single-center design may limit external validity directive to patient population and surgical context [20]. Considering these findings and limitations, several clinical implications and recommendations can be derived. For patients without contraindications, single-entity or adjunct ketorolac in the immediate postoperative period can significantly relieve pain and decrease the need for further opioid pain management; however, clinicians should always use the lowest effective dose for the shortest effective length of time and screen for NSAID contraindications. Paracetamol is also an alternative or adjunctive option particularly in the setting of GI, renal or bleeding risks. The practical approach is to design a multimodal regimen tailored to the needs of a given patient that also combines agents with complementary mechanisms (e.g. paracetamol + NSAID ± regional anesthetic) to optimize analgesia while minimizing opioid exposure. Future research should aim to address some of the important unanswered questions posed by our study: larger, multi-centre randomized trials within surgical categories; assessment of standardized measures of cumulative opioid usage; evaluation of functional recovery endpoints (time to ambulation, length of stay, patient-reported outcomes); and safety measures over longer time intervals including renal function and bleeding outcomes. Other analyses could assess cost considerations and subgroup evaluations of older adults, recipients of renal replacement therapy, or those at high-risk for bleeding. In the end, in this trial, ketorolac administered intravenously, produced better early postoperative analgesia and decreased rescue opioid demand as compared to paracetamol, all with a tolerable side effect profile. These data support inclusion of ketorolac for consideration in multimodal analgesic regimens in the appropriate patient, with caveats of individualized risk stratification and monitoring.

## Conclusion:

Intravenous ketorolac provided superior postoperative analgesia compared with intravenous paracetamol, as evidenced by lower pain scores and reduced rescue analgesia requirement. Both agents were generally well tolerated, though ketorolac showed a slightly higher incidence of mild gastrointestinal effects. Thus, we show the preferential use of ketorolac as part of multimodal pain management in suitable patients, while highlighting the importance of individualized drug selection based on safety considerations.
